# A regional program evaluation of the Stanford Chronic Pain Self-Management Program in Eastern Ontario, Canada

**DOI:** 10.1080/24740527.2024.2440338

**Published:** 2025-01-24

**Authors:** E. Hum, S. Karunananthan, A. Adil, I. Moroz, R. Davidson, C. Liddy

**Affiliations:** a School of Interdisciplinary Health Sciences, University of Ottawa, Ottawa, Ontario, Canada; b Bruyère Research Institute, Ottawa, Ontario, Canada; c Chatham Kent Health Alliance, Chatham, Ontario, Canada; d Chronic Disease Self-Management, Living Healthy Champlain, Ottawa, Ontario, Canada; e Department of Family Medicine, University of Ottawa, Ottawa, Ontario, Canada; f Ontario eConsult Centre of Excellence, The Ottawa Hospital, Ottawa, Ontario, Canada

**Keywords:** Pain clinics, chronic pain, patient participation, self-management, pain management, program evaluation

## Abstract

**Background:**

Health care providers often struggle to treat patients with chronic pain. One potential solution is to facilitate access to programs and tools that develop patients’ skills and confidence in managing their own care.

**Aims:**

This study aimed to describe the uptake of the Chronic Pain Self-Management Program (CPSMP) in Eastern Ontario and evaluate the effectiveness of the program in the acquisition of knowledge, confidence, and skills required to manage chronic pain, as measured by the Patient Activation Measure (PAM).

**Methods:**

Using data routinely collected through the CPSMP between December 2017 and May 2023, we conducted a descriptive analysis of the number of participants each year, their gender, and their age distributions. We conducted a longitudinal analysis of the change in PAM score between participants’ first (baseline) and last (follow-up) day in the program.

**Results:**

Overall, 1023 individuals enrolled in the CPSMP during the study period, with enrollments peaking in 2018 and remaining stable thereafter. There was a higher proportion of females compared to males (69%, *n* = 709) and 50- to 59-year-olds compared to other ages. Of the 1023 participants enrolled, 151 completed PAM surveys at baseline and follow-up (15%), of which 69% experienced an increase of at least 4 points on the PAM (104/151).

**Conclusion:**

Most participants were female and aged 50 to 59 years old. Among a sample of participants with available longitudinal data, the CPSMP demonstrated promising effectiveness at equipping participants with the knowledge, skills, and confidence to manage their pain. Replication in a larger representative sample is warranted.

## RÉSUMÉ

**Contexte :** Les prestataires de soins de santé font souvent face à des difficultés pour traiter les patients souffrant de douleur chronique. Une solution envisageable serait de faciliter l'accès à des programmes et à des outils permettant aux patients d’améliorer leurs compétences et leur confiance en soi, afin de mieux prendre en charge leurs propres soins.

**Objectifs :** Cette étude visait à décrire l'adoption du programme d'autoprise en charge de la douleur chronique (CPSMP) dans l'Est de l'Ontario et à évaluer son efficacité en ce qui concerne l'acquisition des connaissances, la confiance et les compétences nécessaires pour prendre en charge ses propres soins, telles que mesurées par la mesure d’activation du patient (PAM).

**Méthodes :** À partir des données recueillies régulièrement dans le cadre du CPSMP entre décembre 2017 et mai 2023, nous avons réalisé une analyse descriptive du nombre de participants chaque année, de leur sexe et de la répartition de leur âge. Nous avons également mené une analyse longitudinale de l'évolution du score PAM entre le premier (état initial) et le dernier jour (suivi) des participants au programme.

**Résultats :** Dans l'ensemble, 1 023 personnes se sont inscrites au CPSMP au cours de la période d'étude. En outre, les inscriptions ont atteint un sommet en 2018 et sont demeurées stables par la suite. La proportion de femmes était plus élevée que celle des hommes (69 %, n = 709), et les participants âgés de 50 à 59 ans étaient plus nombreux que ceux des autres groupes d’âge. Parmi les 1 023 participants inscrits, 151 ont répondu aux questionnaires PAM au début de l'étude et lors du suivi (15 %), parmi lesquels 69 % ont connu une augmentation d'au moins quatre points par rapport à l'année précédente (104/151).

**Conclusions :** La majorité des participants étaient des femmes âgées de 50 à 59 ans. Parmi un échantillon de participants pour lesquels des données longitudinales étaient disponibles, le CPSMP a démontré une efficacité prometteuse en ce qui concerne l'acquisition des connaissances et de la confiance en soi nécessaires à la prise en charge de la douleur. La reproduction de cette étude sur un échantillon plus large et représentatif est justifiée.

## Introduction

Chronic pain is a highly common issue in primary care. One in five Canadians, including children, lives with chronic pain,^1^ a prevalence similar to reports from other geographical regions: 19% in Europe^2^ and the United States^3^ and 13% in India.^4^ Chronic pain is associated with the greatest reduction in quality of life among chronic diseases,^5^ causing difficulties with working, exercising, sleeping, and doing household chores.^2,3^ Moreover, chronic pain is associated with psychiatric comorbidities, including depression and anxiety.^6–8^ Chronic pain is difficult to treat due to the large number of complex pain cases, the lack of specialist access, and the minimal training of frontline care providers in chronic pain.^9–13^ Given these challenges, it is perhaps not surprising that patients express widespread dissatisfaction with the chronic pain care they receive.^2^

**Figure 1.**
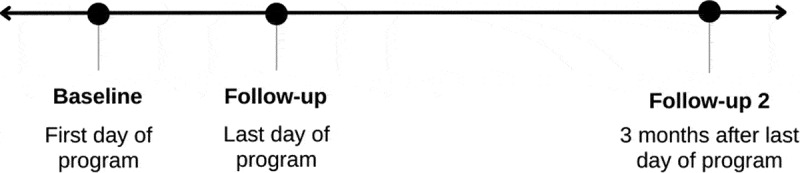
Timeline of Patient Activation Measure survey administration for the Chronic Pain Self-Management Program in the Champlain region of Eastern Ontario. Surveys were administered by Living Healthy Champlain as part of an internal program evaluation.

Chronic pain guidelines recognize that, in addition to the pharmacologic and/or nonpharmacologic treatments that patients receive, patients themselves play a central role in managing their pain and maximizing the benefits of their treatment plan.^1,14–16^ Chronic pain self-management refers to the skills and strategies that an individual employs to limit the impact of their pain in their daily life and to manage their own care.^15^ For example, pain self-management strategies can include goal setting, emotional regulation, and activity pacing.^15^ Several studies indicate that self-management interventions lead to improved treatment outcomes and improved quality of life among patients with chronic pain.^15,17–19^ Because health care providers often struggle to treat patients with chronic pain, one potential solution is to facilitate access to programs that equip patients with skills and confidence in managing their own care.^20–22^

One of the most prominent of such services is the Stanford Chronic Disease Self-Management Program, a 6-week peer-led course developed in the early 1990s by Kate Lorig.^23^ The program is based on self-efficacy theory, which posits that patients with various chronic conditions could benefit from a common intervention through confidence building, skills mastery, modeling, reinterpretation, and social persuasion.^24^ The program has been demonstrated to help patients increase health behaviors (e.g., exercise), reduce negative symptoms associated with their conditions (e.g., disability, fatigue), improve communication with physicians, and avoid hospitalizations.^23–30^ The Stanford Chronic Pain Self-Management Program (CPSMP) was adapted from the Chronic Disease Self-Management Program to tailor more specifically toward patients living with chronic pain.^31^ The program aims to equip participants with the knowledge, skills, and confidence to manage their pain. Though a few studies have assessed the CPSMP’s effectiveness, the results have been mixed.^31,32^ Further evaluation is thus warranted.

In November 2012, a program called Living Healthy Champlain (LHC) launched the CPSMP in Eastern Ontario. LHC collected data on enrollment, participant demographic characteristics, and the effectiveness of the CPSMP as part of an internal program evaluation.

Using these data, in the present study, we describe the uptake of the CPSMP in a health region of Ontario and evaluate its effectiveness in improving self-management by equipping participants with the knowledge, confidence, and skills required to manage their chronic pain. Our findings offer insight into the effectiveness of the program in a “real-world” setting and will be of interest to groups considering implementing the CPSMP or other chronic disease self-management support services in their own jurisdictions.

## Methods

### Design

This study used a cross-sectional design to describe the uptake of the CPSMP during the study period and a longitudinal design to assess its effectiveness in the acquisition of knowledge, confidence, and skills required to manage chronic pain, measured by the change in a participant’s level of patient activation. Patient activation refers to a participant’s involvement in their care, their health behaviors, and their knowledge of their condition. The data analyzed in this study were collected by the program implementation team, LHC, as part of an internal program evaluation.

### Context

The Chronic Disease Self-Management Program was launched in 2009 in Eastern Ontario, Canada, by LHC, which aimed to deliver self-management support and tools to patients across Eastern Ontario. All LHC programs are provided at no cost to participants and are supported by several partner organizations across our health region, including Family health teams, hospitals, community health centers, and community support service agencies. After a successful pilot period,^33^ the Ontario Ministry of Health and Long-Term Care offered funding for the program’s self-management programming. LHC began to expand to other regions across Ontario and develop additional programs targeting specific conditions. In response to increased demands for services among people living with chronic pain, LHC launched the CPSMP in Eastern Ontario in November 2012.

### Intervention

The CPSMP is a highly interactive, peer-led program consisting of weekly 2½-h sessions spread out over 6 weeks. The program has a standardized curriculum and delivery format. The weekly sessions include workshops on sleep, managing difficult emotions, exercise, relaxation techniques, managing fatigue, decision making, problem-solving, communication, treatment evaluation, and creating concrete action plans.^34^ All workshops are led by two volunteer peer leaders who live with chronic pain themselves and/or are caregivers. Peer leaders act as mentors throughout the program, providing peer support to their groups. Clinicians are not involved in the CPSMP, because the program does not offer specific medical advice, guidance, or expertise. Though some peer leaders are clinicians themselves, they do not function in their professional role at any point during the program. All leaders receive at minimum 32 h of standardized training led by two master trainers. Standardized training for leaders was developed by Dr. Kate Lorig and her team at the Self-Management Resource Center.^35^ Leaders are required to lead at least two workshops within the first calendar year following training and one workshop per calendar year thereafter. The program was offered primarily in-person until March 11, 2020. The program was offered primarily online from March 11, 2020, to May 10, 2022, due to the COVID-19 pandemic. Following May 10, 2022, the program was offered both in-person and online.

### Setting

The CPSMP is offered by LHC in the Champlain region of Eastern Ontario, Canada, which has a population of 1.46 million people, the majority of whom live in the city of Ottawa and surrounding suburbs.

### Participants

The CPSMP targeted individuals with chronic pain in the health region. Any individual aged 18 or older with pain could register for the program, without needing a referral or formal diagnosis from a health care practitioner.

### Outcomes

LHC measured patient activation as the primary outcome in their program evaluation of the CPSMP. Patient activation refers to a patient’s participation in their care, health behaviors, and knowledge of their condition. Patient activation was measured using the Patient Activation Measure (PAM), a reliable and validated 13-question survey that places participants on a patient activation scale between 0 and 100.^36^ Higher PAM scores suggest higher levels of patient activation and are associated with significantly better overall health; lower rates of doctor, hospital, and emergency room visits; and increased likelihood of engaging in behaviors to improve overall health and manage specific conditions.^36–39^ Previous research indicates that a difference of 4 points on the PAM is considered clinically meaningful, because it is generally the difference in score between patients who engage in healthy behaviors and those who do not.^40^ Multiple studies have used this value as the minimal clinically important difference to evaluate the effectiveness of their intervention.^41–44^ Participants are also classified into one of four activation levels based on their PAM score^45,46^:

1. Level 1: “disengaged and overwhelmed” (PAM score 0.0–47.0)
2. Level 2: “becoming aware, but still struggling” (PAM score 47.1–55.1)
3. Level 3: “taking action” (PAM score 55.2–72.4)
4. Level 4: “maintaining behaviors and pushing further” (PAM score 72.5–100)

As participants progress through the levels, they have greater odds of experiencing positive outcomes.^47^ Participants in levels 3 and 4, compared to levels 1 and 2, have been shown to have better self-reported quality of life, increased healthy behaviors, and better satisfaction with their health.^47,48^ The PAM has proven to be an effective measure of patient activation.^38,49,50^ Under the direction of the Ontario Ministry of Health and Long-Term Care, LHC is the PAM license holder for the province of Ontario. In 2015, LHC adopted PAM as a provincial measure for programs supporting patient activation. PAM is now the preferred metric for measuring patient activation province-wide.

### Data Collection

LHC collected data on program uptake (who enrolled in the program, when, where, and in what format) on the day of registration. Specifically, LHC collected demographic data (age and gender) and registration data (year of registration, location, and in-person vs. online program format). Participants had the option to not provide information on their age and gender. For the purposes of this study, we differentiated between registrations with postal codes located within urban Eastern Ontario and those located outside of urban Eastern Ontario.

Participants were asked to read and sign a consent form on the first day of the program. LHC collected PAM scores using a survey completed by participants on the first day of the program, at baseline, on the last day of the program, at follow-up, and 3 months after the last day of the program, at follow-up 2 (see Figure 1). Peer leaders collected the PAM surveys at baseline and follow-up and delivered them to LHC staff, who then assigned a unique de-identified code to each participant. Three months following the date of the last workshop, participants were e-mailed or mailed the third and final survey (follow-up 2). They were then asked to mail or e-mail their completed surveys back to LHC. Participants could decline to take part in the survey at any time. Uptake data and PAM data were not linked during the data collection process.

### Ethics Statement

A quality improvement ethics exemption was obtained from the Bruyère Research Ethics Board.

### Data Analysis

LHC provided us with uptake and patient activation data collected between December 2017 and May 2023. First, to describe the uptake of the program, we conducted a descriptive analysis of the number and proportion of participants registered each year, as well as their gender, location, and age distribution. Second, to analyze the effectiveness of the program in the acquisition of knowledge, confidence, and skills required to manage chronic pain, we conducted longitudinal descriptive analyses of the change in PAM score from baseline to follow-up. Specifically, we calculated the proportion of participants who achieved an increase of 4 points on the PAM, based on previous literature that has established this value as the minimal clinically important difference for increases in healthy behaviors.^40–44^ Further, we calculated the proportion of participants in each PAM level at baseline and follow-up. We did not conduct statistical testing in the context of this study. We defined “follow-up” as the second PAM survey completed by a participant, irrespective of the time between a participant’s baseline and follow-up survey completion. Due to poor response rates at follow-up 2 (*n* = 31, 20.5% of the participants who completed a baseline PAM), this time point was not considered in our analysis. PAM data were further stratified according to program format (in-person vs. online). All analyses were conducted using Excel. One participant completed two PAM surveys on the same day at the follow-up time point. For this participant, we calculated the average of the two PAM scores for inclusion in our analysis.

## Results

### CPSMP Usage

**Figure 2.**
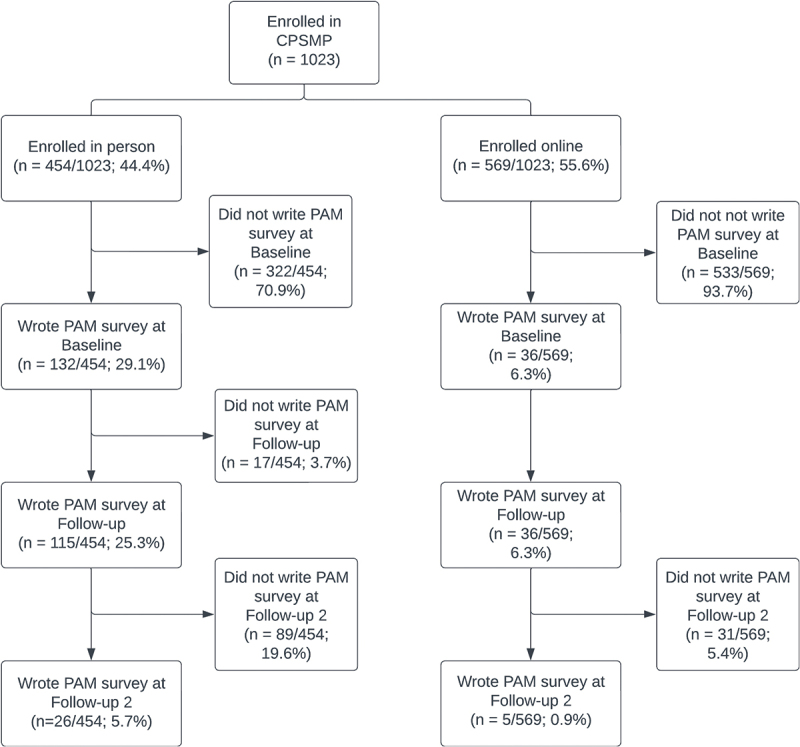
Flowchart of participants enrolled in the Chronic Pain Self-Management Program between December 2017 and May 2023 in the Champlain region of Eastern Ontario. Follow-up 2 was not included in the analysis of the program due to low response rates.

**Figure 3.**
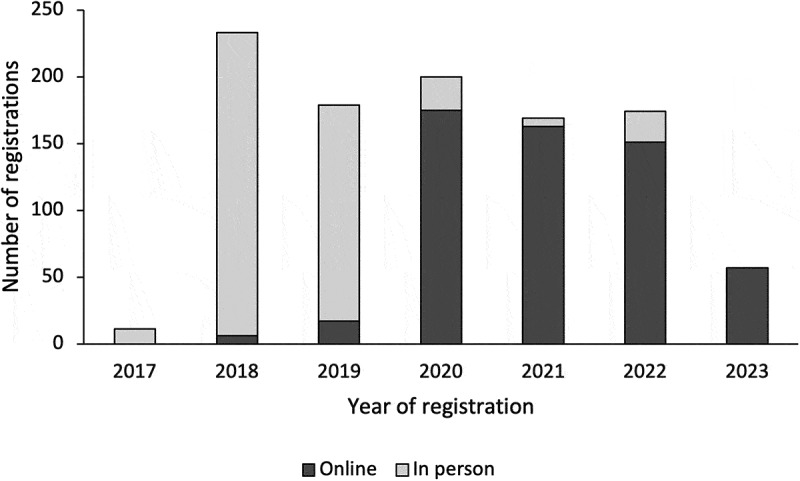
In-person and online registration in the CPSMP between December 2017 and May 2023 in the Champlain region of Eastern Ontario.

A total of 1023 participants enrolled in the CPSMP between December 2017 and May 2023, of whom 454 enrolled in-person and 569 enrolled online (see Figure 2). Participation in the CPSMP peaked in 2018 and remained stable from 2019 to 2022 (see Figure 3). There was a greater proportion of 50- to 59-year-olds (17.7%) compared to other age groups, a greater proportion of female participants compared to male participants (68.6%, *n* = 702), and a greater proportion of participants located within urban Eastern Ontario compared to other locations (70.3%, *n* = 719). Approximately 41.2% (*n* = 421) of participants did not disclose their age, and 15.3% (*n* = 157) did not disclose their gender. A summary of participant characteristics can be found in Table 1.

**Table 1.** Characteristics of participants enrolled in the Chronic Pain Self-Management Program between December 2017 and May 2023.

	In person (*n* = 454), *n* (%)	Online (*n* = 569), *n* (%)	All participants (*n* = 1023), *n* (%)
Age			
20–29 years	6 (1.3)	19 (3.3)	25 (2.4)
30–39 years	17 (3.7)	50 (8.8)	67 (6.5)
40–49 years	38 (8.4)	67 (11.8)	105 (10.3)
50–59 years	68 (15.0)	113 (19.9)	181 (17.7)
60–69 years	43 (9.5)	86 (15.1)	129 (12.6)
70 years and up	49 (10.8)	46 (8.1)	95 (9.3)
Unknown	233 (51.3)	188 (33.0)	421 (41.2)
Gender			
Female	296 (65.2)	406 (71.4)	702 (68.6)
Male	84 (18.5)	74 (13.0)	158 (15.4)
Non-binary	0 (0)	4 (0.7)	4 (0.4)
Prefer to self-describe	0 (0)	2 (0.4)	2 (0.2)
Unknown	74 (16.3)	83 (14.6)	157 (15.3)
Year of registration			
2017	11 (2.4)	0 (0)	11 (1.1)
2018	227 (50.0)	6 (1.1)	233 (22.8)
2019	162 (35.7)	17 (3.0)	179 (17.5)
2020	25 (5.5)	175 (30.8)	200 (19.6)
2021	6 (1.3)	163 (28.6)	169 (16.5)
2022	23 (5.1)	151 (26.5)	174 (17.0)
2023	0 (0)	57 (10.0)	57 (5.6)
Location			
Urban Eastern Ontario	343 (75.6)	376 (66.1)	719 (70.3)
Outside of urban Eastern Ontario	111 (24.4)	193 (33.9)	304 (29.7)

When stratifying participants according to in-person and online program format, the proportion of participants aged 70 and over was higher in person (10.8%, *n* = 49) than online (8.1%, *n* = 46). Interestingly, the proportion of female participants was lower in person (65.2%, *n* = 296) compared to online (71.4%, *n* = 406). Moreover, the proportion of participants outside urban Eastern Ontario was lower in person (24.4%, *n* = 111) than online (33.9%, *n* = 193).

### Effectiveness of the CPSMP

**Figure 4.**
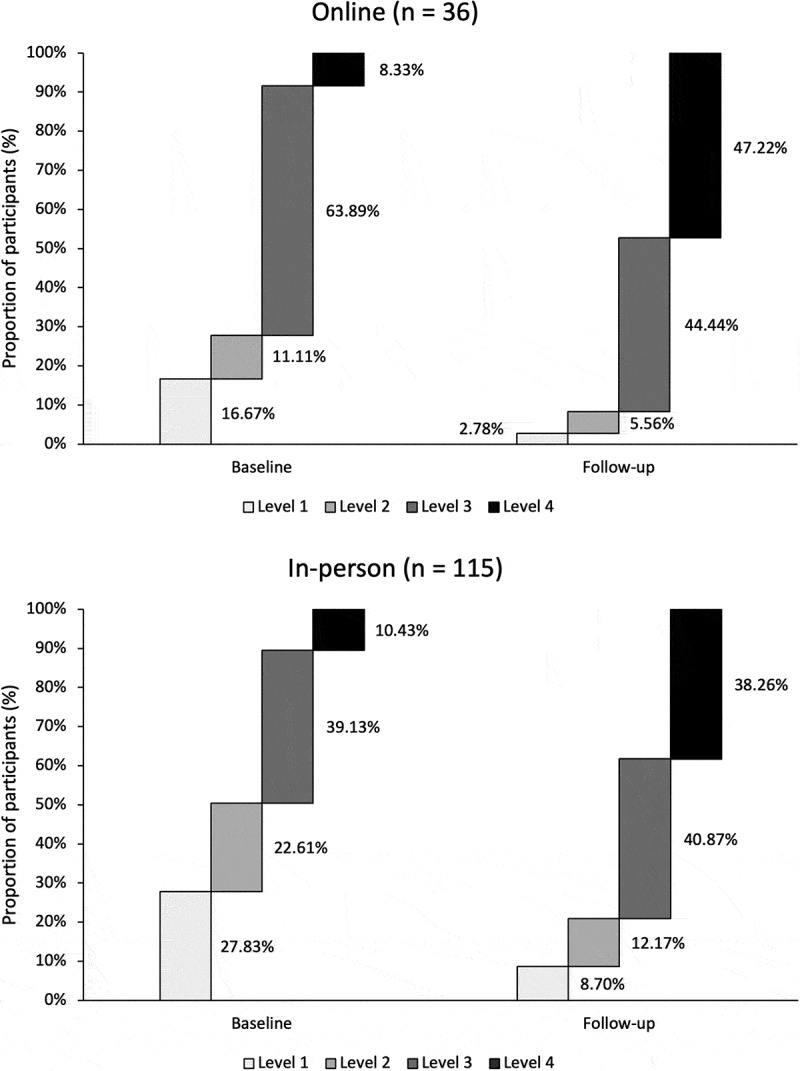
Change in PAM levels from baseline to follow-up, online and in person (*n* = 151), among participants enrolled in the Chronic Pain Self-Management Program in the Champlain region of Eastern Ontario from December 2017 to May 2023. The time to follow-up ranged from 23 days to 14 months and 30 days after baseline. However, 96.0% (145/151) of follow-ups were completed 1 to 2.99 months after baseline.

Of the 1023 participants enrolled in the program, a sample of 168 participants (36 online and 132 in-person) agreed to complete the PAM at baseline (see Figure 2). Of these, 89.9% (*n* = 151) completed the PAM at follow-up. Approximately 96.0% (*n* = 145) of follow-ups were completed between 1 and 3 months after baseline. Overall, 68.9% (*n* = 104) of participants who completed a survey at baseline and follow-up demonstrated an increase of at least 4 points on the PAM. Interestingly, the program’s effectiveness in changing participants’ PAM scores remained similar when stratifying participants according to in-person and online formats. The proportion of participants in PAM levels 1 to 3 decreased from baseline to follow-up, whereas the proportion of those in PAM level 4 increased (see Figure 4). A similar trend was observed when stratifying the data by in-person and online format (see Figure 4).

## Discussion

The CPSMP demonstrated promising results in improving participants’ knowledge, confidence, and skills required to manage their chronic pain, as measured by the PAM. Among a limited sample of participants for whom longitudinal data were available, most experienced improvements of at least 4 points on the PAM from baseline to follow-up. This remained true when stratifying participants by in-person and online program format. Moreover, the proportion of participants in the highest activation level, PAM level 4, increased considerably from baseline to follow-up. Overall, registration in the program peaked in 2018 and remained fairly steady throughout the study period.

LHC used PAM scores as the primary measure of the effectiveness of the CPSMP in the acquisition of knowledge, confidence, and skills required to manage chronic pain. Patient activation is defined as a patient’s ability to manage their health.^36^ The PAM was first developed by Hibbard et al.^36^ as a tool to measure patient activation, with the goal of individualizing care plans to each patient’s level of activation. The existing literature has indicated that the PAM is a reliable and valid tool and that higher PAM scores are associated with increased healthy behavior, greater medication adherence, greater disease-specific knowledge, and a higher likelihood of performing self-management.^51–53^ For this reason, the PAM has been extensively used in developing and evaluating self-management programs.^54–63^ For example, a randomized controlled trial (RCT) compared differences in PAM scores between the intervention and control groups to measure the effectiveness of a web-based self-management program for patients with cardiovascular disease.^55^ Moreover, several studies have compared pre- and post-program PAM scores to measure the impact of self-management programs on self-efficacy and patient activation.^54,57,60,61,63^ Similarly, the present program evaluation study compared participants’ PAM scores before and after program completion to evaluate the effectiveness of the CPSMP in the acquisition of knowledge, confidence, and skills required to manage chronic pain.

Very few studies have analyzed the effects of the Stanford CPSMP. The existing literature has reported a mixture of significant,^31,61,64,65^ and nonsignificant findings.^32,61^ The original RCT that provided the evidence base for the program randomly assigned 110 participants (mean age 40 years, 75% female) in Newfoundland, Canada, to a CPSMP intervention group or a 3-month waitlist control group.^31^ Overall, the study found short-term improvements in self-reported pain, dependency, vitality, life satisfaction, and self-efficacy in the intervention group compared to the control group 3 months posttreatment. Likewise, a pilot study of a remote version of the CPSMP in Ohio found significant changes in pain, depression, and self-efficacy among the 81 program completers (mean age 73.3, 88.8% female) 7 weeks after the start of the program.^64^ The remote program involved sending a material tool kit to participants and conducting weekly scripted phone calls with peer facilitators to reach participants from underserved communities with limited internet access. Another study evaluating the outcomes of a peer-led CPSMP in rural regions of New York found that among the 239 participants who completed the workshop (mean age 64 years, 74.9% female), the program was effective at improving short-term pain self-efficacy, pain disability, depression, and patient activation at 6 months post-program.^61^ However, Though participants demonstrated improvements in patient activation between the first and last days of the program, improvements were not sustained 6 months post-program. A prospective study of the CPSMP in Denmark found significant improvements in pain level, disability, catastrophizing, depression, anxiety, and health worry at the 5-month follow-up among the 87 patients who participated in the program (mean age 52 years, 85% female).^65^ However, when the investigators conducted an RCT of the same Danish lay-led CPSMP among 424 participants (mean age 54, 72% women), they found no significant impacts on pain-related disability, self-efficacy, pain catastrophizing, or health expenditure at the 5-month follow-up and only found small positive effects on emotional distress and illness worry.^32^ Though this mixture of results suggests that more research is needed to assess the long-term impacts of the CPSMP on patient activation and health outcomes, there remains sufficient evidence to suggest that such programs have the potential for positive impact and merit continued implementation.

The data analyzed in the present study were initially collected by LHC as part of an internal program evaluation of the CPSMP. This led to some limitations related to the scope and availability of information in the data. First, participants self-enrolled in the CPSMP on a voluntary basis. It is possible that participants who chose to self-enroll in the program were systematically different from those who did not, which introduces the potential for self-selection bias. Second, participation in the program evaluation component of the CPSMP was optional, and only 16% of all respondents enrolled in the program completed a PAM at baseline. This low response rate introduces the potential for nonresponse bias. Additionally, uptake data, including demographic and registration information, were not linked to PAM data. As a result, we were unable to determine whether PAM respondents differed systematically from nonrespondents. The low response rates and potential for biases limit the generalizability of our findings. Future studies should collect data on the characteristics of respondents and nonrespondents to evaluate potential nonresponse bias.^66^ Third, data collection for follow-up 2 (3 months post-program) relied on mailed or e-mailed surveys. This resulted in important losses to follow-up at follow-up 2, and we were unable to include this time point in our analysis. Low response rates and significant losses to follow-up are common challenges in program evaluation research.^67^ For example, a pre–post effectiveness evaluation of the Chronic Disease Self-Management Program reported response rates ranging from 20% to 85% across different program settings.^68^ Other studies have documented similar issues in nonresponse rates and losses to follow-up.^69,70^ Several strategies can be implemented to improve response rates, including telephone prompting, frequent reminders, and following up with nonrespondents.^66,67,71,72^

Fourth, due to data limitations, we were unable to disaggregate PAM scores according to demographic factors, such as gender or age, to determine whether the program affected certain demographic groups differently. Gender and age differences in program effectiveness could be explored in future research. Fifth, due to the short-term follow-up period in this study, we cannot comment on how effective the program was at maintaining long-term improvements in patient activation. Future studies with longer-term follow-ups are warranted. Sixth, LHC did not collect data on participant attendance, which prevented us from determining the dose of the program received by each participant. As a result, it was not possible to elucidate a dose–response relationship for the program. Finally, due to this study’s nonexperimental design, it is difficult to make a strong causal inference between the CPSMP and patient activation outcomes. Despite these limitations, program evaluation research is integral to providing insight into the effectiveness of health programs, informing decisions about future program development and implementation, and identifying areas for program improvement.^73,74^ The current study provides valuable insight into the uptake and effectiveness of the CPSMP in a real-world setting. The findings from this study provide a strong incentive for future research on the CPSMP using more robust methods.

## Conclusion

Most participants enrolled in the CPSMP between December 2017 to May 2023 in Eastern Ontario were female, aged 50 to 59 years old, and living in urban regions of Eastern Ontario. Our study suggests that, in a real-world setting, the CPSMP is a promising intervention that can potentially improve the knowledge, confidence, and skills required for persons living with chronic pain to manage their condition. Future research on large representative samples is needed to better understand the short- and long-term effectiveness of the program.

## Supplementary Material

CPSMP CJP Manuscript_26Nov24_track changes.docx

## Data Availability

The data that support the findings of this study are available from the corresponding author upon reasonable request.
