# Supplementary material for: A regional program evaluation of the Stanford Chronic Pain Self-Management Program in Eastern Ontario, Canada
Source: Can J Pain. 2025 Jan 24;9(1):2440338. doi: 10.1080/24740527.2024.2440338 (PMC11776463; doi:10.1080/24740527.2024.2440338)
Supplement: CPSMP CJP Manuscript_26Nov24_track changes.docx [file UCJP_A_2440338_SM1469.docx]

A regional program evaluation of the Stanford Chronic Pain Self-Management Program in Eastern Ontario, Canada

E. Hum^a,b^, S. Karunananthan^a,b^, A. Adil^c^, I. Moroz^b^, R. Davidson^d^, and C. Liddy^b,e,f^*

^a^School of Interdisciplinary Health Sciences, University of Ottawa, Ottawa, Canada; ^b^Bruyère Research Institute, Ottawa, Ontario, Canada; ^c^Chatham Kent Health Alliance, Chatham, Ontario, Canada; ^d^Chronic Disease Self-Management, Living Healthy Champlain, Ottawa, Canada; ^e^Department of Family Medicine, University of Ottawa, Ottawa, Canada; ^f^Ontario eConsult Centre of Excellence, The Ottawa Hospital, Ottawa, Canada

*Corresponding author:

Dr. Clare Liddy, Department of Family Medicine, University of Ottawa, Ottawa, Canada, [cliddy@uottawa.ca](mailto:cliddy@uottawa.ca)

ORCiDs:

Sathya Karunananthan: <https://orcid.org/0000-0002-4247-4752>

Clare Liddy: <https://orcid.org/0000-0003-0699-5494>

**Word count**: 3446

**Number of tables**: 1

**Number of figures**: 4

A regional program evaluation of the Stanford Chronic Pain Self-Management Program in Eastern Ontario, Canada

**Background**. Healthcare providers often struggle to treat patients with chronic pain. One potential solution is to facilitate access to programs and tools that develop patients' skills and confidence in managing their own care.

**Aims**. This study aimed to describe the uptake of the Chronic Pain Self-Management Program (CPSMP) in Eastern Ontario and evaluate the effectiveness of the program in the acquisition of knowledge, confidence, and skills required to manage chronic pain, as measured by the Patient Activation Measure (PAM).

**Methods**. Using data routinely collected through the CPSMP between December 2017 and May 2023, we conducted a descriptive analysis of the number of participants each year, their gender, and age distributions. We conducted a longitudinal analysis of the change in PAM score between participants' first (baseline) and last (follow-up) day in the program.

**Results**. Overall, 1023 individuals enrolled in the CPSMP during the study period, with enrolments peaking in 2018 and remaining stable thereafter. There was a higher proportion of females (69%, n=709) compared to males and 50-59-year-olds, compared to other ages. Of the 1023 participants enrolled, 151 (15%) completed PAM surveys at baseline and follow-up, of which 69% (104/151) experienced an increase of at least four points on the PAM scale.

**Conclusion. The majority of participants were female and aged 50-59-years-old. Amongst a sample of participants with available longitudinal data, the** CPSMP demonstrated promising effectiveness at equipping participants with the knowledge, skills, and confidence to manage their pain. Replication in a larger representative sample is warranted.

**Keywords**: pain clinics, chronic pain, patient participation, self-management, pain management, program evaluation

# Introduction

Chronic pain is a highly common issue in primary care. One in five Canadians, including children, lives with chronic pain,^1^ a prevalence similar to reports from other geographical regions: 19% in Europe,^2^ and the United States,^3^ and 13% in India.^4^ Chronic pain is associated with the greatest reduction in quality of life among chronic diseases,^5^ causing difficulties with working, exercising, sleeping, and doing household chores.^2,3^ Moreover, chronic pain is associated with psychiatric comorbidities, including depression and anxiety.^6-8^ Chronic pain is difficult to treat due to the large number of complex pain cases, the lack of specialist access, and the minimal training of frontline care providers in chronic pain.^9-13^ Given these challenges, it is perhaps not surprising that patients express widespread dissatisfaction with the chronic pain care they receive.^2^

Chronic pain guidelines recognize that, in addition to the pharmacologic and/or non-pharmacologic treatments that patients receive, patients themselves play a central role in managing their pain and maximizing the benefits of their treatment plan.^1,14-16^ Chronic pain self-management refers to the skills and strategies that an individual employs to limit the impact of their pain in their daily life and to manage their own care.^15^ For example, pain self-management strategies can include goal setting, emotional regulation, and activity pacing.^15^ Several studies indicate that self-management interventions lead to improved treatment outcomes and improved quality of life among patients with chronic pain.^15,17-19^ Since healthcare providers often struggle to treat patients with chronic pain, one potential solution is to facilitate access to programs that equip patients with skills and confidence in managing their own care.^20-22^

One of the most prominent of such services is the Stanford Chronic Disease Self-Management Program, a six-week peer-led course developed in the early 1990s by Kate Lorig.^23^ The program is based on Self Efficacy theory, which posits that patients with various chronic conditions could benefit from a common intervention through confidence building, skills mastery, modelling, reinterpretation, and social persuasion.^24^ The program has been demonstrated to help patients increase health behaviors (e.g., exercise), reduce negative symptoms associated with their conditions (e.g., disability, fatigue), improve communication with physicians, and avoid hospitalizations.^23-30^ The Stanford Chronic Pain Self-Management Program (CPSMP) was adapted from the Chronic Disease Self-Management Program to tailor more specifically towards patients living with chronic pain.^31^ The program aims to equip participants with the knowledge, skills, and confidence to manage their pain. While a few studies have assessed the CPSMP's effectiveness, the results have been mixed.^31,32^ Further evaluation is thus warranted.

In November 2012, a program called Living Healthy Champlain (LHC) launched the CPSMP in Eastern Ontario. LHC collected data on enrolment, participant demographics, and the effectiveness of the CPSMP as part of an internal program evaluation.

Using this data, in the present study, we describe the uptake of the CPSMP in a health region of Ontario and evaluate its effectiveness in improving self-management by equipping participants with the knowledge, confidence, and skills required to manage their chronic pain. Our findings offer insight into the effectiveness of the program in a “real-world” setting and will be of interest to groups considering implementing the CPSMP or other chronic disease self-management support services in their own jurisdictions.

# Methods

## Design

This study used a cross-sectional design to describe the uptake of the CPSMP during the study period, and a longitudinal design to assess its effectiveness in the acquisition of knowledge, confidence, and skills required to manage chronic pain, measured by the change in a participants’ level of patient activation. Patient activation refers to a participant’s involvement in their care, their health behaviors, and their knowledge of their condition. The data analyzed in this study was collected by the program implementation team, LHC, as part of an internal program evaluation.

## Context

In Eastern Ontario, Canada, the Chronic Disease Self-Management Program was launched in 2009 by LHC, which aimed to deliver self-management support and tools to patients across Eastern Ontario. All LHC programs are provided at no cost to participants and are supported by several partner organizations across our health region, including Family Health Teams, hospitals, Community Health Centers, and Community Support Service Agencies. After a successful pilot period,^33^ the Ontario Ministry of Health and Long-Term Care offered funding for the program's self-management programming. LHC began to expand to other regions across Ontario and develop additional programs targeting specific conditions. In response to increased demands for services amongst people living with chronic pain, LHC launched the CPSMP in Eastern Ontario in November 2012.

## Intervention

The CPSMP is a highly interactive, peer-led program consisting of weekly 2 ½ hour sessions spread out over six weeks. The program has a standardized curriculum and delivery format. The weekly sessions include workshops on sleep, managing difficult emotions, exercise, relaxation techniques, managing fatigue, decision-making, problem-solving, communication, treatment evaluation, and creating concrete action plans.^34^ All workshops are led by two volunteer Peer Leaders who live with chronic pain themselves and/or are caregivers. Peer Leaders act as mentors throughout the program, providing peer support to their groups. Clinicians are not involved in the CPSMP, as the program does not offer specific medical advice, guidance, or expertise. While some Peer Leaders are clinicians themselves, they do not function in their professional role at any point during the program. All Leaders receive at minimum 32 hours of standardized training led by two Master Trainers. Standardized training for leaders was developed by Dr. Kate Lorig and her team at the Self-Management Resource Centre.^35^ Leaders are required to lead at least two workshops within the first calendar year following training, and one workshop per calendar year thereafter. The program was offered primarily in-person until March 11, 2020. The program was offered primarily online from March 11, 2020, to May 10, 2022, due to the COVID-19 pandemic. Following May 10, 2022, the program was offered both in-person and online.

## Setting

The CPSMP is offered by LHC in the Champlain region of Eastern Ontario, Canada, which has a population of 1.46 million people, the majority of whom live in the city of Ottawa and surrounding suburbs.

## Participants

The CPSMP targeted individuals suffering from chronic pain in the health region. Any individual aged 18 or older who was suffering from pain could register for the program, without needing a referral or formal diagnosis from a health care practitioner.

## Outcomes

LHC measured patient activation as the primary outcome in their program evaluation of the CPSMP. Patient activation refers to a patient's participation in their care, health behaviors, and knowledge of their condition. Patient activation was measured using the Patient Activation Measure (PAM), a reliable and validated 13-question survey that places participants on a patient activation scale between zero and 100.^36^ Higher PAM scores suggest higher levels of patient activation and are associated with significantly better overall health, lower rates of doctor, hospital, and emergency room visits, and increased likelihood of engaging in behaviors to improve overall health and manage specific conditions.^36-39^ Previous research indicates that a difference of four points on the PAM scale is considered clinically meaningful, as it is generally the difference in score between patients who engage in healthy behaviours and those who do not.^40^ Multiple studies have used this value as the minimal clinically important difference (MCID) to evaluate the effectiveness of their intervention.^41-44^ Participants are also classified into one of four activation levels based on their PAM score:^45,46^

1. Level 1: "disengaged and overwhelmed" (PAM score from 0.0 – 47.0);
2. Level 2: "becoming aware, but still struggling" (PAM score from 47.1 – 55.1);
3. Level 3: "taking action" (PAM score from 55.2 – 72.4);
4. Level 4: "maintaining behaviors and pushing further" (PAM score from 72.5 – 100).

As participants progress through the levels, they have greater odds of experiencing positive outcomes.^47^ Participants in levels 3 and 4, compared to levels 1 and 2, have been shown to have better self-reported quality of life, increased healthy behaviours, and better satisfaction with their health.^47,48^

The PAM survey has proven to be an effective measure of patient activation.^38,49,50^ Under the direction of the Ontario Ministry of Health and Long-Term Care, LHC is the PAM license holder for the province of Ontario. In 2015, LHC adopted PAM as a provincial measure for programs supporting patient activation. PAM is now the preferred metric for measuring patient activation province wide.

## Data Collection

LHC collected data on program uptake (who enrolled in the program, when, where, and in what format) on the day of registration. Specifically, LHC collected demographic data (age and gender) and registration data (year of registration, location, and in-person vs. online program format). Participants had the option to not provide information on their age and gender. For the purposes of this study, we differentiated between registrations with postal codes located within urban Eastern Ontario and those located outside of urban Eastern Ontario.

Participants were asked to read and sign a consent form on the first day of the program. LHC collected PAM scores using a survey completed by participants on the first day of the program, at Baseline, the last day of the program, at Follow-up, and three months after the last day of the program, at Follow-up 2 (See **Figure** **1**). Peer Leaders collected the PAM surveys at Baseline and Follow-up and delivered them to LHC staff, who then assigned a unique de-identified code to each participant. Three months following the date of the last workshop, participants were e-mailed or mailed the third and final survey (Follow-up 2). They were then asked to mail or email their completed surveys back to LHC. Participants could decline to take part in the survey at any time. Uptake data and PAM data were not linked during the data collection process.

[Figure 1 near here]

## Ethics statement

A quality improvement ethics exemption was obtained from the Bruyère Research Ethics Board.

## Data analysis

LHC provided us with uptake and patient activation data collected between December 2017 and May 2023. First, in order to describe the uptake of the program, we conducted a descriptive analysis of the number and proportion of participants registered each year, as well as their gender, location, and age distribution. Second, in order to analyze the effectiveness of the program in the acquisition of knowledge, confidence, and skills required to manage chronic pain, we conducted longitudinal descriptive analyses of the change in PAM score from Baseline to Follow-up. Specifically, we calculated the proportion of participants who achieved an increase of four points on the PAM scale, based on previous literature that has established this value as the MCID for increases in healthy behaviors.^40-44^ Further, we calculated the proportion of participants in each PAM level at Baseline and Follow-up. We did not conduct statistical testing in the context of this study. We defined "Follow-up" as the second PAM survey completed by a participant, irrespective of the time between a participant's baseline and follow-up survey completion. Due to poor response rates at Follow-up 2 (n=31, 20.5% of the participants who completed a baseline PAM survey), this time point was not considered in our analysis. PAM data were further stratified according to program format (in-person vs online). All analyses were conducted using Excel. One participant completed two PAM surveys on the same day at the Follow-up time point. For this participant, we calculated the average of the two PAM scores to include in our analysis.

# Results

## CPSMP usage

A total of 1023 participants enrolled in the CPSMP between December 2017 and May 2023, of which 454 enrolled in-person and 569 enrolled online (see **Figure 2**). Participation in the CPSMP peaked in 2018 and remained stable from 2019-2022 (see **Figure 3**). There was a greater proportion of 50-59-year-olds compared to other age groups, a greater proportion of female participants (68.6%, n=702) compared to male participants, and a greater proportion of participants located within Urban Eastern Ontario (70.3%, n=719) compared to other locations. Approximately 41.2% (n = 421) of participants did not disclose their age, and 15.3% (n = 157) did not disclose their gender. A summary of participant characteristics can be found in **Table 1**.

[Figure 2 and 3 near here], [Table 1 near here]

When stratifying participants according to in-person and online program format,

the proportion of participants aged 70 and up was higher in-person (10.8%, n=49) than online (8.1%, n=46). Interestingly, the proportion of female participants was lower in-person (65.2%, n=296) compared to online (71.4%, n=406). Moreover, the proportion of participants outside urban Eastern Ontario was lower in-person (24.4%, n=111) than online (33.9%, n=193).

## Effectiveness of the CPSMP

Of the 1023 participants enrolled in the program, a sample of 168 participants (36 online and 132 in-person) agreed to complete a PAM survey at Baseline (see **Figure 2**). Of these, 89.9% (n = 151) completed a PAM survey at Follow-up. Approximately 96.0% (n=145) of follow-ups were completed between 1 and 3 months after baseline. Overall, 68.9% (n=104) of participants who completed a survey at Baseline and Follow-up demonstrated an increase of at least four points on the PAM scale. Interestingly, the program's effectiveness in changing participants’ PAM scores remained similar when stratifying participants according to in-person and online formats. The proportion of participants in PAM levels 1-3 decreased from Baseline to Follow-up, while the proportion of those in PAM level 4 increased (see **Figure 4**). A similar trend is observed when stratifying the data by in-person and online format (see **Figure 4**).

[Figure 4 near here]

# Discussion

The CPSMP demonstrated promising results in improving participants’ knowledge, confidence and skills required to manage their chronic pain, as measured by the PAM. Amongst a limited sample of participants for whom longitudinal data was available, the majority experienced improvements of at least four-points on the PAM scale from Baseline to Follow-up. This remained true when stratifying participants by in-person and online program format. Moreover, the proportion of participants in the highest activation level, PAM level 4, increased considerably from baseline to follow-up. Overall, registration in the program peaked in 2018 and remained fairly steady throughout the study period.

LHC used PAM scores as the primary measure of the effectiveness of the CPSMP in the acquisition of knowledge, confidence, and skills required to manage chronic pain. Patient activation is defined as a patient's ability to manage their health.^36^ The PAM was first developed by Hibbard et al.^36^ as a tool to measure patient activation, with the goal of individualizing care plans to each patient's level of activation. The existing literature has indicated that the PAM is a reliable and valid tool, and that higher PAM scores are associated with increased healthy behavior, greater medication adherence, greater disease-specific knowledge, and a higher likelihood of performing self-management.^51-53^ For this reason, the PAM has been extensively used in developing and evaluating self-management programs.^54-63^ For example, a randomized control trial (RCT) compared differences in PAM scores between the intervention and control groups to measure the effectiveness of a web-based self-management program for patients with cardiovascular disease.^55^ Moreover, several studies have compared pre- and post-program PAM scores to measure the impact of self-management programs on self-efficacy and patient activation.^54,57,60,61,63^ Similarly, the present program evaluation study compared participants' PAM scores before and after program completion to evaluate the effectiveness of the CPSMP in the acquisition of knowledge, confidence, and skills required to manage chronic pain.

Very few studies have analyzed the effects of the Stanford CPSMP. The existing literature has reported a mixture of significant,^31,61,64,65^ and non-significant findings.^32,61^ The original RCT that provided the evidence base for the program randomly assigned 110 participants (mean age 40 years, 75% female) in Newfoundland, Canada, to a CPSMP intervention group or a 3-month waitlist control group.^31^ Overall, the study found short-term improvements in self-reported pain, dependency, vitality, life satisfaction, and self-efficacy in the intervention group compared to the control group three months post-treatment. Likewise, a pilot study of a remote version of the CPSMP in Ohio found significant changes in pain, depression, and self-efficacy among the 81 program completers (mean age 73.3, 88.8% female) seven weeks after the start of the program.^64^ The remote program involved sending a material tool kit to participants and conducting weekly scripted phone calls with peer facilitators to reach participants from underserved communities with limited internet access. Another study evaluating the outcomes of a peer-led CPSMP in rural regions of New York found that among the 239 participants who completed the workshop (mean age 64 years, 74.9% female), the program was effective at improving short-term pain self-efficacy, pain disability, depression, and patient activation at six months post-program.^61^ However, while participants demonstrated improvements in patient activation between the first and last day of the program, improvements were not sustained six months post-program. A prospective study of the CPSMP in Denmark found significant improvements in pain level, disability, catastrophizing, depression, anxiety, and health worry at the five-month follow-up among the 87 patients who participated in the program (mean age 52 years, 85% female).^65^ However, when the investigators conducted an RCT of the same Danish lay-led CPSMP among 424 participants (mean age 54, 72% women), they found no significant impacts on pain-related disability, self-efficacy, pain catastrophizing or health expenditure at the 5-month follow-up, and only found small positive effects on emotional distress, and illness worry.^32^ While this mixture of results suggests that more research is needed to assess the long-term impacts of the CPSMP on patient activation and health outcomes, there remains sufficient evidence to suggest that such programs have the potential for positive impact and merit continued implementation.

The data analyzed in the present study was initially collected by LHC as part of an internal program evaluation of the CPSMP. This led to some limitations related to the scope and availability of information in the data. First, participants self-enrolled in the CPSMP on a voluntary basis. It is possible that participants who chose to self-enrol in the program were systematically different from those who did not, which introduces the potential for self-selection bias. Second, participation in the program evaluation component of the CPSMP was optional, and only 16% of all respondents enrolled in the program completed a PAM survey at Baseline. This low response rate introduces the potential for nonresponse bias. Additionally, uptake data, including demographic and registration information, was not linked to PAM data. As a result, we were unable to determine if PAM respondents differed systematically from nonrespondents. The low response rates and potential for biases limit the generalizability of our findings. Future studies should collect data on the characteristics of respondents and nonrespondents to evaluate potential nonresponse bias.^66^ Third, data collection for Follow-up 2 (three months post-program) relied on mailed or e-mailed surveys. This resulted in important losses to follow-up at Follow-up 2, and we were unable to include this timepoint in our analysis. Low response rates and significant losses to follow-up are common challenges in program evaluation research.^67^ For example, a pre-post effectiveness evaluation of the Chronic Disease Self-Management Program reported response rates ranging from 20-85% across different program settings.^68^ Other studies have documented similar issues in nonresponse rates and losses to follow-up.^69,70^ Several strategies can be implemented to improve response rates, including telephone prompting, frequent reminders, and following-up with non-respondents.^66,67,71,72^

Fourth, due to data limitations, we were unable to disaggregate PAM scores according to demographic factors, such as gender or age, to determine if the program affected certain demographic groups differently. Gender- and age-differences in program effectiveness could be explored in future research. Fifth, due to the short-term follow-up period in this study, we cannot comment on how effective the program was at maintaining long-term improvements in patient activation. Future studies with longer-term follow-ups are warranted. Sixth, LHC did not collect data on participant attendance, which prevented us from determining the dose of the program received by each participant. As a result, it was not possible to elucidate a dose-response relationship for the program. Finally, due to this study's non-experimental design, it is difficult to make a strong causal inference between the CPSMP and patient activation outcomes. Despite these limitations, program evaluation research is integral to providing insight into the effectiveness of health programs, informing decisions about future program development and implementation, and identifying areas for program improvement.^73,74^ The current study provides valuable insight into the uptake and effectiveness of the CPSMP in a real-world setting. The findings from this study provide a strong incentive for future research on the CPSMP using more robust methods.

# Conclusion

The majority of participants enrolled in the CPSMP between December 2017 to May 2023 in Eastern Ontario were female, aged 50-59-years-old, and living in urban regions of Eastern Ontario. Our study suggests that, in a real-world setting, the CPSMP is a promising intervention that can potentially improve the knowledge, confidence, and skills required for persons living with chronic pain to manage their condition. Future research on large representative samples is needed to better understand the short- and long-term effectiveness of the program.

# Acknowledgements

The authors wish to thank the providers and patients who use LHC.

# Disclosure statement

Authors have no conflicts of interest to declare.

# Funding information

Funding for this project was provided through the Canadian Institutes of Health Research. The authors affirm their independence from the funder. The funder played no part in the study design, collection, analysis, or interpretation of the data, in the writing of the report, or in the decision to submit the article for publication. All authors had full access to all the data in the study and can take responsibility for the integrity of the data and the accuracy of the data analysis. Living Healthy Champlain (LHC) receives program funding from Ontario Health East to provide the Chronic Pain Self-Management Program in Eastern Ontario.

# Data availability statement

The data that support the findings of this study are available from the corresponding author, C.L., upon reasonable request.

# References

1. An action plan for pain in Canada. 2021. Ottawa, Ontario: Health Canada.

2. Breivik H, Collett B, Ventafridda V, Cohen R, Gallacher D. 2006. Survey of chronic pain in Europe: Prevalence, impact on daily life, and treatment. Eur J Pain. 10(4):287–333. doi:10.1016/j.ejpain.2005.06.009

3. Kennedy J, Roll JM, Schraudner T, Murphy S, McPherson S. 2014. Prevalence of Persistent Pain in the U.S. Adult Population: New Data From the 2010 National Health Interview Survey. J Pain. 15(10):979–984. doi:10.1016/j.jpain.2014.05.009

4. Dureja GP, Jain PN, Shetty N, Mandal SP, Prabhoo R, Joshi M, Goswami S, Natarajan KB, Iyer R, Tanna DD, et al. 2014. Prevalence of Chronic Pain, Impact on Daily Life, and Treatment Practices in India. Pain Pract. 14(2):E51–E62. doi:10.1111/papr.12132

5. Choinière M, Dion D, Peng P, Banner R, Barton PM, Boulanger A, Clark AJ, Gordon AS, Guerriere DN, Guertin M, et al. 2010. The Canadian STOP-PAIN project - Part 1: Who are the patients on the waitlists of multidisciplinary pain treatment facilities? Can J Anesth. 57:539–548. doi:10.1007/s12630-010-9305-5

6. Dominick CH, Blyth FM, Nicholas MK. 2012. Unpacking the burden: Understanding the relationships between chronic pain and comorbidity in the general population. PAIN. 153(2):293–304. doi:10.1016/j.pain.2011.09.018

7. Ligthart L, Visscher CM, Van Houtem CMHH, Geels LM, Vink JM, De Jongh A, Boomsma DI. 2014. Comorbidity Among Multiple Pain Symptoms and Anxious Depression in a Dutch Population Sample. J Pain. 15(9):945–955. doi:10.1016/j.jpain.2014.06.007

8. Gore M, Sadosky A, Stacey BR, Tai K-S, Leslie D. 2012. The Burden of Chronic Low Back Pain: Clinical Comorbidities, Treatment Patterns, and Health Care Costs in Usual Care Settings. Spine. 37(11):E668–E677. doi:10.1097/BRS.0b013e318241e5de

9. Onishi E, Kobayashi T, Dexter E, Marino M, Maeno T, Deyo RA. 2017. Comparison of Opioid Prescribing Patterns in the United States and Japan: Primary Care Physicians’ Attitudes and Perceptions. J Am Board Fam Med. 30(2):248–254. doi:10.3122/jabfm.2017.02.160299

10. Penney LS, Ritenbaugh C, DeBar LL, Elder C, Deyo RA. 2016. Provider and patient perspectives on opioids and alternative treatments for managing chronic pain: a qualitative study. BMC Fam Pract. 17(1):164. doi:10.1186/s12875-016-0566-0

11. Upshur CC, Luckmann RS, Savageau JA. 2006. Primary care provider concerns about management of chronic pain in community clinic populations. J Gen Intern Med. 21(6):652–655. doi:10.1111/j.1525-1497.2006.00412.x

12. O’Rorke JE, Chen I, Genao I, Panda M, Cykert S. 2007. Physicians’ Comfort in Caring for Patients with Chronic Nonmalignant Pain. Am J Med Sci. 333(2):93–100. doi:10.1097/00000441-200702000-00005

13. Watt-Watson J, McGiIIion M, Hunter J, Choiniere M, Clark AJ, Dewar A, Johnston C, Lynch M, Morley-Forster P, Moulin D, et al. 2009. A survey of prelicensure pain curricula in health science faculties in Canadian universities. Pain Res Manage.:439–44. doi:10.1155/2009/307932

14. Korownyk CS, Montgomery L, Young J, Moore S, Singer AG, MacDougall P, Darling S, Ellis K, Myers J, Rochford C, et al. 2022. PEER simplified chronic pain guideline: Management of chronic low back, osteoarthritic, and neuropathic pain in primary care. Can Fam Physician. 68(3):179–190. doi:10.46747/cfp.6803179

15. Nicholas MK, Blyth FM. 2016. Are Self-Management Strategies Effective in Chronic Pain Treatment? Pain Manag. 6(1):75–88. doi:10.2217/pmt.15.57

16. Von Korff M, Gruman J, Schaefer J, Curry SJ, Wagner EH. 1997. Collaborative Management of Chronic Illness. Ann Intern Med. 127(12):1097–1102. doi:10.7326/0003-4819-127-12-199712150-00008

17. Blyth FM, March LM, Nicholas MK, Cousins MJ. 2005. Self-management of chronic pain: a population-based study. Pain. 113(3):285–292. doi:10.1016/j.pain.2004.12.004

18. Damush TM, Kroenke K, Bair MJ, Wu J, Tu W, Krebs EE, Poleshuck E. 2016. Pain self‐management training increases self‐efficacy, self‐management behaviours and pain and depression outcomes. Eur J Pain. 20(7):1070–1078. doi:10.1002/ejp.830

19. Solberg Nes L, Børøsund E, Varsi C, Eide H, Waxenberg LB, Weiss KE, Morrison EJ, Støle HS, Kristjansdottir ÓB, Bostrøm K, et al. 2024. Living well with chronic pain: a 12-month randomized controlled trial revealing impact from the digital pain self-management program EPIO. PAIN Rep. 9(4):e1174. doi:10.1097/PR9.0000000000001174

20. Hylands-White N, Duarte RV, Raphael JH. 2017. An overview of treatment approaches for chronic pain management. Rheumatol Int. 37(1):29–42. doi:10.1007/s00296-016-3481-8

21. Hestmann R, Bratås O, Grønning K. 2023. Chronic pain self-management interventions in primary care – does it make any difference? A qualitative study. :1–10. doi:10.1186/s12913-023-09548-8

22. Bodenheimer T, MacGregor K, Sharifi C. 2005. Helping Patients Manage Their Chronic Conditions. Oakland CA: California Healthcare Foundation.

23. Lorig KR, Sobel DS, Stewart AL, Brown BW, Bandura A, Ritter P, Gonzalez VM, Laurent DD, Holman HR. 1999. Evidence Suggesting That a Chronic Disease Self-Management Program Can Improve Health Status While Reducing Hospitalization: A Randomized Trial. Med Care. 37(1):5–14.

24. Lorig K. 2014. Chronic disease self-management program: insights from the eye of the storm. Front Public Health. 2:253. doi:10.3389/fpubh.2014.00253

25. Ory MG, Ahn S, Jiang L, Smith ML, Ritter PL, Whitelaw N, Lorig K. 2013. Successes of a National Study of the Chronic Disease Self-Management Program: Meeting the Triple Aim of Health Care Reform. Med Care. 51(11):992–998.

26. Lorig KR, Hurwicz M-L, Sobel D, Hobbs M, Ritter PL. 2005. A national dissemination of an evidence-based self-management program: a process evaluation study. Patient Educ Couns. 59(1):69–79. doi:10.1016/j.pec.2004.10.002

27. Franek J. 2013. Self-Management Support Interventions for Persons With Chronic Disease: An Evidence-Based Analysis. Ont Health Technol Assess Ser. 13(9):1–60.

28. Lorig KR, Ritter PL, Laurent DD, Plant K. 2006. Internet-Based Chronic Disease Self-Management: A Randomized Trial. Med Care. 44(11):964–971.

29. Ory MG, Smith ML, Ahn S, Jiang L, Lorig K, Whitelaw N. 2014. National Study of Chronic Disease Self-Management: Age Comparison of Outcome Findings. Health Educ Behav. 41(1_suppl):34S-42S. doi:10.1177/1090198114543008

30. Lorig KR, Sobel DS, Ritter PL, Laurent D, Hobbs M. 2001. Effect of a self-management program on patients with chronic disease. Eff Clin Pract ECP. 4(6):256–262.

31. LeFort SM, Gray-Donald K, Rowat KM, Jeans ME. 1998. Randomized controlled trial of a community-based psychoeducation program for the self-management of chronic pain. Pain. 74(2):297–306. doi:10.1016/S0304-3959(97)00190-5

32. Mehlsen M, Hegaard L, Ørnbøl E, Jensen JS, Fink P, Frostholm L. 2017. The effect of a lay-led, group-based self-management program for patients with chronic pain: a randomized controlled trial of the Danish version of the Chronic Pain Self-Management Programme. Pain. 158(8):1437–1445. doi:10.1097/j.pain.0000000000000931

33. Liddy C, Johnston S, Guilcher S, Irving H, Hogel M, Jaglal S. 2015. Impact of a chronic disease self-management program on healthcare utilization in eastern Ontario, Canada. Prev Med Rep. 2:586–590. doi:10.1016/j.pmedr.2015.07.001

34. Living Healthy Champlain - Living A Healthy Life With Chronic Pain. [accessed 2023 Sep 17]. https://www.livinghealthychamplain.ca

35. Trainings Offered by SMRC. SMRC - Self-Manag Resour Cent [Internet]. [accessed 2024 Nov 25]. https://selfmanagementresource.com/training/

36. Hibbard JH, Stockard J, Mahoney ER, Tusler M. 2004. Development of the Patient Activation Measure (PAM): Conceptualizing and Measuring Activation in Patients and Consumers: Development of the Patient Activation Measure (PAM). Health Serv Res. 39(4p1):1005–1026. doi:10.1111/j.1475-6773.2004.00269.x

37. Peters AE, Keeley EC. 2017. Patient Engagement Following Acute Myocardial Infarction and Its Influence on Outcomes. Am J Cardiol. 120(9):1467–1471. doi:10.1016/j.amjcard.2017.07.037

38. Prey JE, Qian M, Restaino S, Hibbard J, Bakken S, Schnall R, Rothenberg G, Vawdrey DK, Masterson Creber R. 2016. Reliability and validity of the patient activation measure in hospitalized patients. Patient Educ Couns. 99(12):2026–2033. doi:10.1016/j.pec.2016.06.029

39. Gleason KT, Tanner EK, Boyd CM, Saczynski JS, Szanton SL. 2016. Factors associated with patient activation in an older adult population with functional difficulties. Patient Educ Couns. 99(8):1421–1426. doi:10.1016/j.pec.2016.03.011

40. Hibbard J, Greene J, Tusler M. 2009. Improving the Outcomes of Disease Management by Tailoring Care to the Patient’s Level of Activation. Am J Manag Care. 15(6):353–60.

41. John JR, Tannous WK, Jones A. 2020. Outcomes of a 12-month patient-centred medical home model in improving patient activation and self-management behaviours among primary care patients presenting with chronic diseases in Sydney, Australia: a before-and-after study. BMC Fam Pract. 21(1):158. doi:10.1186/s12875-020-01230-w

42. Anderson JK, Wallace LM. 2018. Evaluation of uptake and effect on patient-reported outcomes of a clinician and patient co-led chronic musculoskeletal pain self-management programme provided by the UK National Health Service. Br J Pain. 12(2):104–112. doi:10.1177/2049463717734015

43. Tan KK, Chan SW-C, Wang W, Vehviläinen-Julkunen K. 2016. A salutogenic program to enhance sense of coherence and quality of life for older people in the community: A feasibility randomized controlled trial and process evaluation. Patient Educ Couns. 99(1):108–116. doi:10.1016/j.pec.2015.08.003

44. Schuit AS, Holtmaat K, Lissenberg-Witte BI, Eerenstein SEJ, Zijlstra JM, Eeltink C, Becker-Commissaris A, Van Zuylen L, Van Linde ME, Menke-van Der Houven Van Oordt CW, et al. 2022. Efficacy of the eHealth application Oncokompas, facilitating incurably ill cancer patients to self-manage their palliative care needs: A randomized controlled trial. Lancet Reg Health - Eur. 18:100390. doi:10.1016/j.lanepe.2022.100390

45. Dammery G, Vitangcol K, Ansell J, Ellis LA, Smith CL, Carrigan A, Braithwaite J, Zurynski Y. 2023. The Patient Activation Measure (PAM) and the pandemic: Predictors of patient activation among Australian health consumers during the COVID-19 pandemic. Health Expect. 26(3):1107–1117. doi:10.1111/hex.13725

46. Insignia Health - Activate Your Population. Insign Health [Internet]. [accessed 2023 Jun 27]. https://www.insigniahealth.com/

47. Greene J, Hibbard JH, Sacks R, Overton V, Parrotta CD. 2015. When Patient Activation Levels Change, Health Outcomes And Costs Change, Too. Health Aff (Millwood). 34(3):431–437. doi:10.1377/hlthaff.2014.0452

48. Hernar I, Graue M, Igland J, Richards DA, Riise HKR, Haugstvedt A, Kolltveit B-CH. 2023. Patient activation in adults attending appointments in general practice: a cross-sectional study. BMC Prim Care. 24(1):144. doi:10.1186/s12875-023-02102-9

49. Kosar C, Besen DB. 2019. Adaptation of a patient activation measure (PAM) into Turkish: reliability and validity test. Afr Health Sci. 19(1):1811–1820. doi:10.4314/ahs.v19i1.58

50. Lightfoot CJ, Wilkinson TJ, Memory KE, Palmer J, Smith AC. 2021. Reliability and Validity of the Patient Activation Measure in Kidney Disease: Results of Rasch Analysis. Clin J Am Soc Nephrol CJASN. 16(6):880–888. doi:10.2215/CJN.19611220

51. Mosen DM, Schmittdiel J, Hibbard J, Sobel D, Remmers C, Bellows J. 2007. Is Patient Activation Associated With Outcomes of Care for Adults With Chronic Conditions? J Ambulatory Care Manage. 46(4):306–314. doi:10.1097/JAC.0000000000000477

52. Hibbard JH, Greene J. 2013. What The Evidence Shows About Patient Activation: Better Health Outcomes And Care Experiences; Fewer Data On Costs. Health Aff (Millwood). 32(2):207–214. doi:10.1377/hlthaff.2012.1061

53. Hendriks M, Rademakers J. 2014. Relationships between patient activation, disease-specific knowledge and health outcomes among people with diabetes; a survey study. BMC Health Serv Res. 14(1):393. doi:10.1186/1472-6963-14-393

54. Dorn SD, Palsson OS, Woldeghebriel M, Fowler B, McCoy R, Weinberger M, Drossman DA. 2015. Development and pilot testing of an integrated, web‐based self‐management program for irritable bowel syndrome (IBS). Neurogastroenterol Motil. 27(1):128–134. doi:10.1111/nmo.12487

55. Engelen MM, Van Dulmen S, Puijk-Hekman S, Vermeulen H, Nijhuis-van Der Sanden MW, Bredie SJ, Van Gaal BG. 2020. Evaluation of a Web-Based Self-Management Program for Patients With Cardiovascular Disease: Explorative Randomized Controlled Trial. J Med Internet Res. 22(7):e17422. doi:10.2196/17422

56. Gholami M, Abdoli Talaei A, Tarrahi MJ, Taqi FM, Galehdar N, Pirinezhad P. 2021. The effect of self-management support program on patient activation and inner strength in patients with cardiovascular disease. Patient Educ Couns. 104(12):2979–2988. doi:10.1016/j.pec.2021.04.018

57. Gomaa S, Posey J, Bashir B, Mallick AB, Vanderklok E, Schnoll M, Zhan T, Wen K-Y. 2023. Feasibility of a Text Messaging–Integrated and Chatbot-Interfaced Self-Management Program for Symptom Control in Patients With Gastrointestinal Cancer Undergoing Chemotherapy: Pilot Mixed Methods Study. JMIR Form Res. 7:e46128. doi:10.2196/46128

58. Hibbard JH, Greene J. 2014. The Impact of an Incentive on the Use of an Online Self-Directed Wellness and Self-Management Program. J Med Internet Res. 16(10):e217. doi:10.2196/jmir.3239

59. Hosseinzadeh H, Verma I, Gopaldasani V. 2020. Patient activation and Type 2 diabetes mellitus self-management: a systematic review and meta-analysis. Aust J Prim Health. 26(6):431–442. doi:10.1071/PY19204

60. Krouse RS, Grant M, McCorkle R, Wendel CS, Cobb MD, Tallman NJ, Ercolano E, Sun V, Hibbard JH, Hornbrook MC. 2016. A chronic care ostomy self‐management program for cancer survivors. Psychooncology. 25(5):574–581. doi:10.1002/pon.4078

61. Pullyblank K, Brunner W, Scribani M, Krupa N, Wyckoff L, Strogatz D. 2022. Evaluation of a Peer Led Chronic Pain Self-Management Program in a Rural Population. J Prim Care Community Health. 13:21501319221121464. doi:10.1177/21501319221121464

62. Young L, Hertzog M, Barnason S. 2016. Effects of a home-based activation intervention on self-management adherence and readmission in rural heart failure patients: the PATCH randomized controlled trial. BMC Cardiovasc Disord. 16(1):176. doi:10.1186/s12872-016-0339-7

63. Fløde M, Iversen MM, Aarflot M, Haltbakk J. 2017. Lasting impact of an implemented self‐management programme for people with type 2 diabetes referred from primary care: a one‐group, before–after design. Scand J Caring Sci. 31(4):789–795. doi:10.1111/scs.12398

64. Sheth K, Ritter PL, Lorig K, Steinman L, FallCreek S. 2022. Remote Delivery of the Chronic Pain Self-management Program Using Self-directed Materials and Small-group Telephone Support: A Pilot Study. J Appl Gerontol. 41(5):1329–1335. doi:10.1177/07334648211062805

65. Mehlsen M, Heegaard L, Frostholm L. 2015. A prospective evaluation of the Chronic Pain Self-Management Programme in a Danish population of chronic pain patients. Patient Educ Couns. 98(5):677–680. doi:10.1016/j.pec.2015.01.008

66. Statistics Canada. 2015. Response and nonresponse. Stat Can [Internet]. [accessed 2024 Oct 4]. https://www150.statcan.gc.ca/n1/pub/12-539-x/2009001/response-reponse-eng.htm

67. Booker QS, Austin JD, Balasubramanian BA. 2021. Survey strategies to increase participant response rates in primary care research studies. Fam Pract. 38(5):699–702. doi:10.1093/fampra/cmab070

68. Hevey D, Wilson O’Raghallaigh J, O’Doherty V, Lonergan K. 2020. Pre-post effectiveness evaluation of Chronic Disease Self-Management Program (CDSMP) participation on health, well-being and health service utilization. Chronic Illn. 16(2):146–158. doi:10.1177/1742395318792063

69. Hanewinkel R, Wiborg G. 2006. Initial evaluation of a real-world self-help smoking cessation programme for adolescents and young adults. Addict Behav. 31(10):1939–1945. doi:10.1016/j.addbeh.2005.12.022

70. Hapidou EG, Pham E, Bartley K, Anthonypillai J, Altena S, Patterson L, Zacharias R. 2021. Chronic pain program management outcomes: Long-term follow-up for Veterans and civilians. J Mil Veteran Fam Health. 7(S2):74–91. doi:10.3138/jmvfh-2021-0054

71. Day NA, Dunt DR, Day S. 1995. Maximizing Response to Surveys in Health Program Evaluation At Minimum Cost Using Multiple Methods: Mail, Telephone, and Visit. Eval Rev. 19(4):436–450. doi:10.1177/0193841X9501900405

72. Treasury Board of Canada Secretariat. 1998. Program evaluation methods : measurement and attribution of program results. Ottawa: Public Affairs Branch.

73. Bowen S. 2012. A Guide to Evaluation in Health Research - CIHR [Internet]. [place unknown]: Canadian Institutes of Health Research; [accessed 2024 Oct 4]. https://cihr-irsc.gc.ca/e/45336.html

74. Adams J, Neville S. 2020. Program Evaluation for Health Professionals: What It Is, What It Isn’t and How to Do It. Int J Qual Methods. 19:160940692096434. doi:10.1177/1609406920964345

# Tables with captions

**Table 1**. Characteristics of participants enrolled in the Chronic Pain Self-Management Program between December 2017 and May 2023.

|  | In-person  (n=454) | Online  (n=569) | All participants  (n=1023) |
| --- | --- | --- | --- |
| Age |  |  |  |
| 20-29 years | 6 (1.3%) | 19 (3.3%) | 25 (2.4%) |
| 30-39 years | 17 (3.7%) | 50 (8.8%) | 67 (6.5%) |
| 40-49 years | 38 (8.4%) | 67 (11.8%) | 105 (10.3%) |
| 50-59 years | 68 (15.0%) | 113 (19.9%) | 181 (17.7%) |
| 60-69 years | 43 (9.5%) | 86 (15.1%) | 129 (12.6%) |
| 70 years and up | 49 (10.8%) | 46 (8.1%) | 95 (9.3%) |
| Unknown | 233 (51.3%) | 188 (33.0%) | 421 (41.2%) |
| Gender |  |  |  |
| Female | 296 (65.2%) | 406 (71.4%) | 702 (68.6%) |
| Male | 84 (18.5%) | 74 (13.0%) | 158 (15.4%) |
| Non-binary | 0 (0%) | 4 (0.7%) | 4 (0.4%) |
| Prefer to self-describe | 0 (0%) | 2 (0.4%) | 2 (0.2%) |
| Unknown | 74 (16.3%) | 83 (14.6%) | 157 (15.3%) |
| Year of registration |  |  |  |
| 2017 | 11 (2.4%) | 0 (0%) | 11 (1.1%) |
| 2018 | 227 (50.0%) | 6 (1.1%) | 233 (22.8%) |
| 2019 | 162 (35.7%) | 17 (3.0%) | 179 (17.5%) |
| 2020 | 25 (5.5%) | 175 (30.8%) | 200 (19.6%) |
| 2021 | 6 (1.3%) | 163 (28.6%) | 169 (16.5%) |
| 2022 | 23 (5.1%) | 151 (26.5%) | 174 (17.0%) |
| 2023 | 0 (0%) | 57 (10.0%) | 57 (5.6%) |
| Location |  |  |  |
| Urban Eastern Ontario | 343 (75.6%) | 376 (66.1%) | 719 (70.3%) |
| Outside of Urban Eastern Ontario | 111 (24.4%) | 193 (33.9%) | 304 (29.7%) |

# Figure captions

**Figure 1**. Timeline of Patient Activation Measure survey administration for the Chronic Pain Self-Management program in the Champlain region of Eastern Ontario. Surveys were administered by Living Healthy Champlain as part of an internal program evaluation.

**Figure 2**. Flowchart of participants enrolled in the Chronic Pain Self-Management Program between December 2017 and May 2023 in the Champlain region of Eastern Ontario. Abbreviations: CPSMP, Chronic Pain Self-Management Program; PAM, Patient Activation Measure. Follow-up 2 was not included in the analysis of the program due to low response rates.

**Figure 3**. In person and online registration in the CPSMP between December 2017 and May 2023 in the Champlain region of Eastern Ontario.

**Figure 4**. Change in PAM levels from baseline to follow-up, online and in-person (n=151), among participants enrolled in the Chronic Pain Self-Management Program in the Champlain region of Eastern Ontario from December 2017 to May 2023. The time to follow-up ranged from 23 days to 14 months and 30 days after baseline. However, 96.0% (145/151) of follow-ups were completed 1 to 2.99 months after baseline.
